# Potential of Egg as Complementary Food to Improve Nutrient Intake and Dietary Diversity

**DOI:** 10.3390/nu14163396

**Published:** 2022-08-18

**Authors:** Mieke Faber, Linda Malan, Herculina S. Kruger, Hannah Asare, Marina Visser, Tshiphiri Mukwevho, Cristian Ricci, Cornelius M. Smuts

**Affiliations:** 1 Non-Communicable Diseases Research Unit, South African Medical Research Council, Cape Town 7505, South Africa; 2 Centre of Excellence for Nutrition (CEN), North-West University, Potchefstroom 2530, South Africa; 3 Africa Unit for Transdisciplinary Health Research, North-West University, Potchefstroom 2530, South Africa

**Keywords:** egg consumption, egg allergy, dietary intake, dietary diversity, infants

## Abstract

The original aim was to determine the effect of egg consumption on infant growth in a low socioeconomic community in South Africa in a randomized controlled trial. Enrolment was, however, prematurely stopped due to COVID-19 lockdown regulations. The resultant small sample (egg group *n* = 70; control group *n* = 85) was followed up to assess the feasibility of egg consumption for eight months in terms of dietary intake, egg usage and perceived effects of lockdown on child feeding. Egg consumption remained low in the control group, <10% consumed egg ≥4 days/week at the follow-up points. In the egg group, egg was frequently consumed at midpoint (daily 87.1%, 4–6 days 8.1%) and endpoint (daily 53.1%, 4–6 days 21.9%). At endpoint, dietary intake of cholesterol and vitamin D was higher, and intake of niacin and vitamin B6 lower in the egg group compared to the control group. Dietary diversity was low, 36.2% of the egg group and 18.9% of the control group (*p* < 0.05) achieved minimum dietary diversity at endpoint. No babies developed egg allergy or sensitization, and adjusted regression analysis showed that frequency of egg intake was not related with the incidence or duration of allergy-related symptoms. This study showed that frequent egg consumption can contribute safely to complementary food for babies, especially in low- and middle-income countries.

## 1. Introduction

Stunting, which affects 22.2% (149.2 million) of children below five years globally [1], is associated with increased morbidity, mortality, loss of developmental potential, and poor educational performance, as well as increased risks of chronic diseases in adulthood [2,3]. Stunting is caused by, among other, suboptimal breastfeeding, inappropriate complementary feeding practices, inadequate access to or use of different types of food and inadequate intake of micronutrients [4].

Infants are susceptible to growth faltering, particularly between the age of 6 to 12-months, when they are introduced to complementary foods that are nutritionally inadequate and of low energy density [5]. In low- and middle-income countries (LMICs), the complementary diet is often deficient in key micronutrients [6], essential amino acids [7] and essential fatty acids [8], and only one in four children (aged 6–23 months) consume a sufficiently diverse diet [9]. Some evidence further suggests that in some LIMCs, a large proportion of energy intake in 6–24-month-old children is provided by snack foods and sugar-sweetened beverages [10]. South Africa is no exception, with unhealthy foods, such as sugar-sweetened beverages, sugary foods, and salty snacks, being introduced at a very young age, and only 23% of 6–23-month-old children are given a minimum acceptable diet [11]. The prevalence of stunting in children under five in South Africa is estimated at 27% [11].

Micronutrient supplementation and fortification interventions are effective in reducing the burden of undernutrition in children younger than five years in LIMCs [12]. Dietary modification, focusing on dietary diversification and inclusion of micronutrient-rich foods may, however, be a more cost-effective and long-term strategy to improve dietary intake during infancy and early childhood [13]. Animal-source foods, such as eggs, meat, fish, and dairy, are rich sources of multiple highly bioavailable micronutrients [14], and inclusion of animal-source foods as part of the predominantly cereal-based complementary diet in LIMCs is encouraged [7]. Consumption of animal source foods may improve child growth [15], and there is some evidence suggesting that the provision of an egg a day for a period of six months in babies older than six months reduces stunting [16].

Previously, early introduction of eggs and egg-containing foods was avoided because of concerns for egg allergy [17]. Current recommendations, however, support the introduction of egg at the age of six months [18,19,20]. Studies have shown that in 6–23-month-old children, the prevalence of egg consumption is considerably lower in Africa compared to other regions in the world [21], with egg consumption in the richest households being three to five times higher than in the poorest households [22]. In South Africa, egg is one of the cheapest sources of animal-source proteins available to consumers [23,24].

Eggs are a good source of high-quality protein, micronutrients (e.g., choline, iron, zinc, vitamin A, phosphorous, and iodine), and high-quality lipids, such as phospholipids and polyunsaturated fatty acids [8], which are important for optimal brain and immune development [25]. Despite the nutritional benefit of eggs being well documented [16,25,26], there is limited evidence available regarding including egg during the early complementary feeding phase to enhance child growth and development. We, therefore, aimed to study the effect of providing one egg per day for a period of six months, beginning at the age of 6 to <9-months, on the growth, motor development, micronutrient, and morbidity status of infants from a low socioeconomic community in South Africa. However, enrolment into the study was prematurely stopped because of COVID-19 lockdown regulations. This was necessary as the study could not continue as per protocol in terms of home visits to monitor and collect data, as well as participants could not come to the study site for midpoint assessments. We did, however, continue to provide eggs to the babies who were already enrolled and collected information on morbidity symptoms to monitor allergic symptoms. This study is, therefore, viewed as a preliminary study, with the aim to assess the feasibility of daily egg consumption in terms of dietary intake and usage of egg, allergy symptoms, and perceived effects of lockdown on child feeding and care.

## 2. Materials and Methods

### 2.1. Study Population

Study participants resided in the peri-urban Jouberton area in the greater Matlosana Municipality, which is 200 km from the nearest metropolitan area (Johannesburg), South Africa. Health facilities in the area report a monthly birth rate of approximately 250 normal deliveries and 120 babies delivered by cesarean section. About 30 to 40 children per month are admitted to hospital with severe acute malnutrition [27]. The District Health Information System does not capture data on stunting prevalence in South Africa. However, the results of a recent study conducted in this municipality showed a stunting rate of 28.5% at the age of six months [28]. The unemployment rate in the area is 32.7% [27].

### 2.2. Study Design and Participants

The original study was designed as a randomized controlled trial with a parallel design. Trained fieldworkers recruited potential study participants, mainly at the household level but also through the clinics when infants were brought to the clinic for their 14-week vaccination. Infants aged 6 to <9-months and residing in the study area were eligible to take part in the study if their mother or legal guardian was 18 years or older, and was planning to live in the study area for the next nine months, was born as a singleton, had no known allergy or intolerance to egg, had no severe obvious congenital abnormalities, severe acute malnutrition (weight-for-length z-score (WLZ) < −3), severe anaemia (hemoglobin <70 g/L), any disease referred for hospitalization by clinic staff, or was receiving special nutritional supplements as part of feeding programs. Before enrolment and with written informed consent from the mother, eligible infants were screened for acute malnutrition, severe anemia, and egg allergy and sensitization.

Eligible infants whose mothers consented were enrolled in the study when they were at least six-months but not yet nine-months-old. At enrolment, infants were randomly allocated to either the egg or the control group, using a randomization sequence of pseudo-random numbers generated by the RANNOR function of the SAS software package version 9.4. Given the nature of the intervention, it was not possible to blind participants to the respective study group after enrolment, but it was blinded to those involved in the laboratory, anthropometric, and dietary intake assessments.

Participants in the egg group received one large-sized grain-fed egg (grade 1) per day, while participants in the control group received no treatment. Participants in the egg group received a dozen eggs per week; seven eggs for the intervention baby, and the rest for consumption by family members. All eggs were provided by the same supplier for the duration of the study. As an incentive for the control group, the household received 5 kg of maize meal per month during the trial (equivalent to the cost of weekly eggs given to an intervention household), and one month’s supply of a dozen eggs per week after the completion of the study.

The original sample size calculations were based on an expected effect size of higher than 0.3 units for length-for-age z-score (LAZ) and a 50% reduction in stunting prevalence, given a baseline prevalence of 27%. The required sample size was 250 babies per group, allowing for a 25% drop-out rate.

Enrolment started on 13 February, 2020. Enrolment was stopped in March 2020, when strict lockdown regulations were implemented due to COVID-19. At the time, 155 infants had been enrolled, which is the final sample for this preliminary study. The study therefore did not have the power to measure the effect on the main outcome (growth). Moreover, during lockdown, research activities could not proceed as per protocol. Nonetheless, the provision of eggs to the babies who were already enrolled continued, and allergy symptoms were continuously monitored. The planned duration of the intervention was six months. This was, however, extended to eight months due to COVID-19 and the different lockdown levels and restrictions thereby instituted, to enable the study team to do the endpoint assessments as per protocol. Although initially implemented as an RCT, we view this study as a preliminary study, focusing on dietary intake, usage of egg and allergy symptoms. The RCT was started over with a new cohort of 500 babies, and results will be published elsewhere.

### 2.3. Intervention Procedures

Babies in the egg group were exposed to egg if they tested negative for egg sensitization with a skin prick test and, if negative, consumed a small amount of egg, where after they were monitored for allergy symptoms for at least 1.5 h during the baseline visit at the study site. Mothers were informed about allergy symptoms and were given a small booklet with recipe ideas and information on safe storage and handling of eggs. The mother/caregiver was contacted on the second day to assess if the baby had any reaction.

Babies were gradually introduced to eggs during the first two weeks of the trial, whereafter consumption of one egg per day was recommended. Initially, fieldworkers did weekly home visits to deliver eggs and monitor adherence, morbidity, and dietary intake. Due to COVID-19 lockdown regulations, delivery of eggs and data collection could not continue as originally planned. With the assistance of a local NGO, eggs, maize meal, and weekly monitoring forms were delivered to participant homes, and completed forms were picked up monthly, while adhering to all COVID-19 safety rules and regulations. During lockdown, there was no direct contact with the participants, and all monitoring activities were done telephonically. The egg and the control group were treated the same.

### 2.4. Research Procedures and Data Collection

Baseline and endpoint assessments were done at the study site and included anthropometric measurements, blood sampling, and questionnaire data (dietary and allergy data). Socio-demographic information and early breastfeeding and complementary feeding practices were collected by questionnaire at baseline. At midpoint (approximately three months after baseline), questionnaire data (usual consumption of foods and perceived effect of lockdown) were collected telephonically. Morbidity data were collected continuously with a daily diary by the caretaker. Trained fieldworkers interviewed the mother or caregiver (collectively referred to from here on forward as caregivers) in either English or the native language (Setswana) of the area.

*Sociodemographic information* was collected using a structured questionnaire.

*Anthropometric measurements* were taken by fieldworkers who were trained according to the WHO Training Course on Child Growth Assessment for infants [29]. Babies were undressed and weighed to the nearest 0.01 kg using a calibrated digital infant scale (Seca model 354; GmbH & Co. KG., Hamburg, Germany, maximum weight 20 kg). Recumbent length was measured to the nearest 0.1 cm using an infantometer (Seca model 416, GmbH & Co. KG., Hamburg, Germany). All measurements were done in duplicate, and if the first two measurements differed by >0.05 kg for weight or by >0.3 cm for length, a third measurement was done, and the average of the two closest values was used.

Anthropometric indexes (z-scores) were generated using WHO Anthro 2006 software. Wasting was defined as WLZ less than −2 SDs, stunting as LAZ less than −2 SDs, and underweight and overweight as weight-for-age *z* score (WAZ) less than −2 SDs or >2 SDs, respectively.

*Allergy questionnaire and skin prick test:* Clinical allergy symptoms were assessed by the Childhood Allergy and Immunology Research (CAIR) questionnaire, and all babies were tested for egg allergy sensitization with a skin prick test and fed a teaspoon of cooked egg if not sensitized. Acute allergy symptoms within 1.5 h of exposure together with positive skin prick test were used to confirm egg allergy (no tolerance) and subsequent exclusion from the study. A positive skin prick test without clinical symptoms confirmed sensitization with tolerance. Since eggs were to be provided daily, which may cause tolerant sensitization to evolve into allergy, all babies with positive skin prick tests to egg were excluded. Mothers of allergic and sensitized babies were given information on how to handle their sensitized/allergic baby and referred to the pediatric clinic at the local hospital.

*A capillary blood sample* was collected into a lithium heparin Microvette^®^ CB 300 (Sarstedt) by means of a finger and/or heel prick due to the volume of blood needed which is a maximum of 500 µL. Hemoglobin was measured on site using a portable Hb 201 + HemoCue system (HemoCue Angelholm, Sweden) to determine anemia status.

*Morbidity data*: Information on infant morbidity (including allergy symptoms), such as skin rashes, diarrhea, upper respiratory diseases, and episodes of fever, were collected using a daily wellness/illness diary and questionnaire, which, until lockdown started, were collected during weekly home-visits by the field workers. During lockdown, the forms were delivered to participant homes and picked up monthly with the assistance of a local NGO.

*Breastfeeding and complementary feeding practices* were collected retrospectively using a structured questionnaire based on WHO guidelines for assessing infant and young child feeding practices [30].

*Usual consumption of foods* over the past seven days was collected using unquantified food frequency questions that had previously been used and tested for face validity in the study population [31].

*Dietary intake* was determined using a single multiple pass 24-h dietary recall that was administered to the primary caregiver of the infant. Portion sizes were estimated using examples of food (e.g., savory snacks), food containers (e.g., infant foods, yogurt), household utensils, food photographs, and the “dish-up and measure” method. The dietary kit was used to assist the mothers/caregivers in quantifying the amount of food consumed by the infant the previous day. Portion sizes were converted to grams using the South African Medical Research Council (SAMRC) Food Quantities Manual [32]. Breastmilk intake was estimated as 675 mL [33]. All foods and drinks were coded according to the South African Food Composition Database [34]. Food intake data were converted to energy and nutrients using STATA software and the most recent South African food composition database available at the time. Percentage of babies with micronutrient intakes below the estimated average required (EAR) [35,36] was calculated.

The dietary diversity score (DDS) and the proportion of children consuming a diet with minimum dietary diversity (at least five of the eight food groups during the 24-h recall period) [37] were calculated using the 24-h recall food intake data.

### 2.5. Data Analysis

Data were analyzed using IBM SPSS for Windows, version 27 (SPSS Inc., Chicago, IL, USA). Baseline characteristics of study participants are presented as frequencies (categorical data) and means and standard deviations or medians and interquartile ranges (continuous data). Groups were compared using either chi-square test (categorical data) or Mann–Whitney U-test (continuous data). Association of frequency of egg intake with incidence and duration of allergy symptoms within the total group were tested with logistic and linear regression, respectively. The model was adjusted for age, sex, birth weight, frequency of breastfeeding and formula intake, total energy intake, and education level of the caretaker. A *p*-value < 0.05 was considered statistically significant.

### 2.6. Ethics

Ethical approval was obtained from the HREC of NWU (NWU-00452-19-A1). After institutional ethical approval, the study was registered with the National Health Research Database, and approval was sought from the Provincial Department of Health. The community’s approval to conduct the study was obtained through an engagement process with relevant community-based structures. The study was registered at clinicaltrials.gov (NCT05168085). The study was monitored by a data safety monitoring board (DSMB), which consisted of a pediatrician, dietician/nutritionist, and a statistician. Infants identified as having severe anemia (Hb < 70 g/L) and severe malnutrition (WLZ < −3) at each phase of the data collection were to be referred to the clinic for further assessment and treatment. Informed consent was obtained from the mothers of the infants before any study-specific procedures were performed.

Babies with serious adverse events (SAEs) were referred to the Klerksdorp Hospital, Department of Paediatrics. Mothers/caregivers were reimbursed for all travel costs to the central study site if not transported to the study site by the research team.

## 3. Results

In total, 163 infants were screened at baseline, of whom 155 were enrolled (egg group *n* = 70; control group *n* = 85). One infant was excluded because of egg allergy, and two because of egg allergy sensitization as determined through the skin prick test. Of the 155 infants, 148 (95.5%) completed the study. Seven (4.5%) babies dropped out of the study because they relocated (*n* = 5), traveled at the time of the exit survey (*n* = 1), or were lost to follow-up (*n* = 1). For both groups, respectively, the age of the babies was 7.7 ± 0.9 months at baseline and 16.1 ± 1.0 months at follow-up. None of the enrolled babies developed egg allergy or egg allergy sensitization during the 8-month intervention period.

### 3.1. Baseline Characteristics of Study Participants

At baseline, 93.5% of the respondents were the mother of the baby (egg group 92.9%, *n* = 65; control group 94.1%, *n* = 80), and 6.5% a caregiver, of whom all but two stayed in the same household as the baby. Baseline characteristics of the study participants are given in Table 1. At baseline, approximately two-thirds of the babies had already been introduced to egg (egg group 64.3%; control group 70.6%) and were breastfeeding (egg group 64.3%; control group 68.2%).

### 3.2. Dietary Intake

#### 3.2.1. Energy and Nutrient Intake

The 24-h dietary recall was not completed for 11 (7.1%) babies at baseline and 16 (10.8%) babies at endpoint, due to the respondent not being able to recall all foods and drinks consumed during the 24-h recall period as the baby was not in her full-time care the previous day. Dietary data was therefore available for 144 babies (egg group *n* = 63; control group *n* = 81) at baseline, and 132 (egg group *n* = 58; control group *n* = 74) at endpoint.

Energy, macronutrient, and micronutrient intakes, based on 24-h dietary recall data, are presented in Table 2. At baseline, median energy and nutrient intakes did not differ between the two groups, except for magnesium and potassium, which were higher in the control group. At endpoint, nutrient intakes that differed between the two groups were niacin and vitamin B6 (higher in the control group) and cholesterol and vitamin D (higher in the egg group).

At endpoint, 62.1% of babies in the egg group and 14.9% in the control group ate egg on the day of recall. Energy, macronutrient, and micronutrient intakes for those eating egg versus those not eating egg, regardless of the intervention group, are presented in Table 3. Babies who ate egg on the day of recall had higher intakes of total protein, animal protein, total fat, monounsaturated fat, polyunsaturated fat, cholesterol, phosphorous, vitamin B12, pantothenic acid, biotin, vitamin D, and vitamin E.

**Figure 1:**
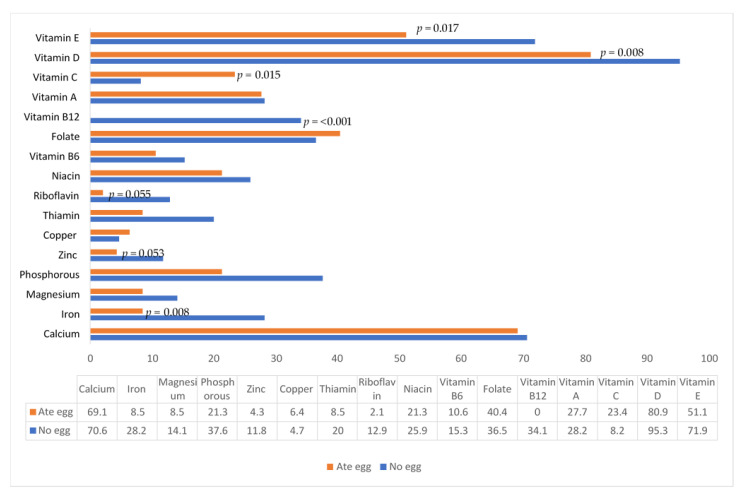
Percentage of babies with intake below the Estimated Average Requirement (EAR) [35,36] for babies who ate egg during the 24-h recall period versus those who did not eat egg, regardless of treatment group.

The percentage of babies with intake below the EAR for babies who ate egg during the 24-h recall period versus those who did not eat egg, regardless of treatment group, is shown in Figure 1. Significantly fewer babies who ate egg during the recall period had inadequate intake of vitamin E, vitamin D, vitamin B12, and iron, while significantly fewer babies who did not eat egg had inadequate intake of vitamin C.

Only two babies had a protein intake below the EAR. The median (25th and 75th percentiles) energy intake, expressed as a percentage of the estimated energy requirement [35], was 128% (96%, 170%) for babies who ate egg during the recall period, and 124% (94%, 148%) for those who did not eat egg.

#### 3.2.2. Food Groups and Dietary Diversity

At endpoint, egg was consumed during the 24-h recall period by 62.1% of babies in the egg group, versus 14.9% in the control group (*p* < 0.001), and double the number of children in the egg group compared to the control group achieved minimum dietary diversity (36.2% versus 18.9%, *p* = 0.026). (Table 4)

#### 3.2.3. Frequency of Foods Consumed

The frequency of consumption during the week preceding baseline, midpoint and endpoint was recorded for selected foods items. Results are presented in Table 5. The midpoint questionnaire was completed per phone for 129 participants. Not all the participants could be reached by phone, explaining the smaller midpoint sample. Shifts in food intake were observed from baseline (mean age 7.7 months) to endpoint (mean age 16.1 months), with no obvious differences between the two groups. The percentage of babies who frequently (at least four days/week) consumed formula milk, pureed baby foods, and infant cereals decreased, while the percentage for maize meal porridge increased. The percentage of babies who frequently ate vegetables or fruit remained very low across the study period. The percentage of babies who consumed animal source at least once a week increased over the study period. Liver was eaten at least once a week by approximately a quarter of the babies at baseline and just under half of the babies at endpoint. At endpoint, approximately a quarter of the babies frequently consumed sweets and salty savory snacks.

### 3.3. Egg Usage and Perceptions on Egg

The frequency of egg consumption during the week preceding baseline, midpoint and endpoint is summarized in Table 6, and preparation and amount consumed for those who ate egg in the previous week are summarized in Table 7. The percentage of babies who frequently consumed eggs remained low in the control group across the study period, with fewer than 10% of babies who consumed egg frequently (at least four days/week) at the respective three time points. In the egg group, egg was frequently consumed by most babies at midpoint (every day 87.1%, 4–6 days 8.1%) and endpoint (every day 53.1%, 4–6 days 21.9%). Egg was eaten either boiled, fried, or scrambled, and was frequently eaten mixed with other food, particularly porridge or potato. In the egg group, egg was eaten mostly one at a time and once a day.

The caregivers’ perceptions regarding the usage of eggs were determined during the exit interview. According to participants in the egg group, they like giving egg to the baby (93.8%), the baby likes to eat egg (85.9%), and eggs are easy to store (93.8%) and prepare (98.5%).

For both groups combined, most participants agreed that eggs are affordable (95.1%) and good for baby’s health (97.9%), and that all eggs are of the same quality (86.4%). When asked whether they agreed that babies can eat raw egg, 52.8% disagreed, 18.0% were undecided, and 29.2% agreed. Five (4.2%) participants said that there were traditional beliefs regarding eating eggs. The benefits and risks of giving eggs to babies are summarized in Table 8.

### 3.4. Effect of Lockdown on Feeding and Care of the Baby

Of the 129 participants who completed the midpoint questionnaire, 45 (34.9%) said that lockdown affected the feeding of the baby, and 14 (10.9%) said that lockdown affected the care of the baby. Feeding of the baby was affected due to a shortage of food [“*Baby’s food finished before month-end*”] and lack of money to buy food [“*Because I don’t have enough money to buy food for the baby*”]. Respondents also reported loss of income due to loss of opportunities for informal vending [“*Because I don’t have any income and we are not free to sell some things*”], loss of opportunities for part-time jobs [“*We have struggled a lot because of lack of money because the father used to go and do piece jobs*”], and loss of salary [“*Because now the father’s salary is half*”].

The effect on the care of the baby was related mostly to the lack of access to routine services at the clinic. Mothers were advised not to go to the clinic [“*Because they said we must not take the baby to the clinic*”], or if they went to the clinic, they were sent home without being attended to [“*The baby had a fever so there was no money to buy flue medicine because at the clinic they returned us*”]. As a result, some of the babies missed their nine months immunization [“*Can’t take the baby to the clinic; they said they are not immunizing*”; “*The clinic sisters returned us; I have also skipped 9 months*”]

### 3.5. Allergy-Related Symptoms

The frequency of egg intake was positively associated with the duration of wheezing in the unadjusted model (β = 0.413, *p* = 0.021), but not in the model adjusted for age, sex, birth weight, frequency of breastfeeding and formula intake, total energy intake and education level of the caretaker (β = −0.155, *p* = 0.580) (Table 9). There were two SAEs during the study, one in each study group. The two SAEs were not related to the study.

## 4. Discussion

Due to premature cessation of enrolment and resultant small sample size, the study was not powered to determine the effect of daily egg consumption on any of the primary outcomes. Despite the small sample size, the results of the study show that frequent egg consumption is feasible in infants. Egg intake was associated with higher protein, cholesterol, and micronutrient intakes, the caregivers were positive towards feeding their babies egg, and the frequency of egg intake was not related to incidence and duration of allergy symptoms.

At endpoint, egg was eaten by 14.9% in the control group during the 24-h recall period. This is very similar to the 12.1% reported for 1–<10-year-old children in a recent study in South Africa [38] and slightly higher than the 8.9% reported for 6–11-months-old infants in Eastern and Southern Africa [22]. Nearly a third of the babies in the egg group did not eat egg during the 24-h recall period at endpoint. Egg was delivered to the households monthly and it is possible that the household ran out of eggs before the endpoint measurements were taken, primarily due to the general lack of food in the household during lockdown. During midpoint, mothers reported loss of income and lack of money to buy food for the household during lockdown. It may, however, also indicate babies becoming fatigued of eating egg daily and a resultant decrease in adherence over the eight-month period.

Although few differences in nutrient intake between the egg and control groups were observed at endpoint, the proportion of babies who consumed at least five of the eight food groups that were used to calculate the dietary diversity score was double in the egg group compared to the control group, suggesting that egg consumption did not replace other foods and had the potential to increase dietary diversity, which is similar to the findings of an egg intervention study that was done in Malawi [39]. Dietary diversity was, however, low, with 36.2% of babies in the egg group and 18.9% in the control group achieving the minimum dietary diversity at endpoint. Low dietary diversity is common in infants and young children in South Africa [40]. Egg consumption should thus be promoted within the context of a diverse diet.

Comparing nutrient intake at endpoint between the egg and control group showed only a few significant differences between the two groups. However, when comparing nutrient intake for babies that consumed egg during the recall period versus those who did not at endpoint, babies who consumed egg had significantly higher intakes of various nutrients, such as animal protein, PU fat, vitamin B12, and vitamin D, all which are critical for child growth and development [41]. None of the children who consumed egg on the day of recall had a vitamin B12 intake below the EAR. On average, 50 g of egg provides 0.8 µg of vitamin B12 [34], which is higher than EAR of 0.7 µg for 1–3-year-old children [35]. Eggs are one of the few foods that naturally contain vitamin D [42]. Although dietary vitamin D intake was significantly higher for children who consumed egg during the recall period versus those who did not, dietary vitamin D intake was inadequate for more than 80% of the children. A study in Malawi showed that a six-month egg intervention increased nutrient intake and nutrient density of the complementary diet for several nutrients, although nutrient deficiencies in the diet remained high [43].

To add variation in providing egg and avoid the baby becoming fatigued of eating egg daily, mothers were encouraged to use different preparation methods and either feed the baby mashed egg as such or mixed with the baby’s food. Egg was eaten either boiled, fried, or scrambled and was frequently eaten mixed with other food, particularly porridge or potato. Mothers were educated and reminded to always prepare the egg well-cooked before feeding their babies.

Infants and young children should not be given raw egg. A 60% higher rate for allergy to raw egg compared to cooked egg has been reported for 12 to 36-month-old children in South Africa [44]. Furthermore, the consumption of raw egg can cause salmonella food poisoning [45]. Just over half of the mothers disagreed that raw egg can be given to the baby, the others were either undecided or agreed. This suggests that a strong education campaign is needed through integrated interventions and social marketing campaigns.

Nearly two-thirds of babies had been introduced to egg before enrolment in the study, indicating that most mothers viewed egg as suitable for infants. However, nearly a third of the mothers thought that eggs should be given to babies before the age of six months, which is not in line with the national and international guidelines, which promote exclusive breastfeeding for the first six months [46]. Early introduction of complementary foods is, however, widespread in South Africa [40].

Traditional beliefs regarding eating eggs do not seem to be a major issue, for instance, in our study, only <5% of participants confirmed this belief. Lutter [39] argued that increasing egg consumption in LIMCs is hampered by economic barriers rather than cultural barriers. However, the majority of mothers in our study found eggs to be affordable. In addition, eggs are considered one of the cheapest animal protein sources available to consumers in South Africa [23,24].

Potential egg allergy in infants may be a concern [47]. A recent South African study showed that between 1 and 2% Black African 12–36-month-old children are allergic to egg depending on urban or rural living conditions [44]. It is encouraging that of the 163 screened infants, only one baby was excluded because of egg allergy and two because of egg allergy sensitization, while none of the enrolled babies developed egg allergy or egg allergy sensitization during the eight-month intervention period. Moreover, the frequency of egg intake was not related to either incidence or duration of allergy symptoms, which is in agreement with the results of a previous study [48]. There is currently consensus that early introduction of eggs as complementary food does not increase the risk of allergy incidence, as reflected in the revised guidelines of the American Academy of Pediatrics in 2008 [49]. A recent systematic review and meta-analysis actually found that early introduction of egg reduced the risk for egg allergy [18].

The results of the study should be interpreted within the limitations of the small size, and self-reported data (dietary intake and morbidity). Furthermore, a 24-h recall is based on memory and portion sizes are estimated. Both under- and over-reporting can thus occur. Although multiple 24-h recalls may have been preferable, the aim of the dietary intake data was to describe the mean (or median) intakes at the group level, in which case a single 24-h recall per participant is acceptable [50]. Breastmilk intake was estimated using average age-specific published values [33], which is common practice in dietary assessment [51,52]. A further limitation is that delivery of eggs to children in the egg group was done monthly, and monitoring adherence was compromised by the strict lockdown regulations due to COVID-19 at the time of the study. Nonetheless, the study provides valuable information on the feasibility of frequent inclusion of egg in the complementary diet of babies.

In conclusion, egg intake was associated with higher protein and micronutrient intakes, and caregivers were positive towards feeding their babies egg. The frequency of egg intake was not related to the incidence or duration of allergy symptoms. This study, therefore, showed that frequent egg consumption can contribute safely to complementary food for babies, especially in LIMCs.

## Figures and Tables

**Table 1:** Baseline characteristics of the 155 participants at enrolment.

Characteristics	Egg Group (*n* = 70)	Control Group (*n* = 85)
*Infant characteristics*		
Sex: Male, *n* (%)	34 (48.6)	44 (51.8)
Female, *n* (%)	36 (51.4)	41 (48.2)
Age, months	7.7 ± 0.9 ^1^	7.7 ± 0.9
Breastfeeding at enrolment, *n* (%)	45 (64.3)	58 (68.2)
Baby already introduced to egg, *n* (%)	45 (64.3)	60 (70.6)
Higher risk for allergy ^2^, *n* (%)	18 (26.1)	15 (18.3)
*Anthropometric status*		
Length-for-age z-score (LAZ)	−1.26 (−1.85, −0.60) ^3^	−1.37 (−2.07, −0.48)
Stunted (<−2 LAZ), *n* (%)	14 (20.0)	25 (29.4)
Weight-for-age z-score (WAZ)	−0.42 (−1.25, 0.17)	−0.61 (−1.51, 0.24)
Underweight (<−2 WAZ), *n* (%)	7 (10.0)	12 (14.1)
Weight-for-length z-score (WLZ)	0.54 (−0.40, 1.30)	0.24 (−0.62, 1.31)
Wasted (<−2 WLZ), *n* (%)	1 (1.4)	4 (4.7)
Overweight (>+2 WLZ), *n* (%)	8 (11.4)	14 (16.5)
Hemoglobin (Hb), g/L	110 ± 13	108 ± 12
Anemic (Hb <110 g/L), *n* (%)	32 (45.7)	42 (49.4)
*Mother /caregiver characteristics*		
Age, y	30.2 ± 9.5	29.3 ± 6.7
Education, ≥Grade 10, *n* (%)	54 (78.3)	71 (83.5)
Married, *n* (%)	8 (11.4)	6 (7.1)
*Household characteristics*		
Electricity at home, *n* (%)	59 (84.3)	75 (88.2)
Tap water at home, *n* (%) ^4^	65 (94.2)	79 (94.0)
Flush toilet at home, *n* (%)	65 (92.9)	74 (87.1)
Number of people in household	5 (4, 7)	6 (4, 7)
Number of child grants per household	2 (1, 3)	2 (2, 3)

^1^ Values are means ± SD (all such values); ^2^ Mother/caregiver reported previous reaction to food, angioedema, hives, eczema or coughing or wheezing without baby having a cold or flu; ^3^ Values are medians, 25th and 75th percentile in parenthesis (all such values); ^4^ Information missing for two households.

**Table 2:** Energy, macronutrient, and micronutrient intakes at baseline and endpoint.

Nutrient	Group ^1^	Baseline Median (P25, P75)	Endpoint Median (P25, P75)
Energy (kcal)	Egg	690 (617, 839)	931 (767, 1090)
	Control	736 (627, 895)	903 (733, 1098)
	*p*-value ^2^	0.143	0.759
Protein (g)	Egg	14.6 (10.5, 16.8)	26.6 (18.1, 35.0)
	Control	15.9 (12.10, 21.6)	23.8 (18.4, 34.8)
	*p*-value	0.138	0.633
Plant protein (g)	Egg	3.6 (2.0, 5.7)	6.9 (51, 10.8)
	Control	4.3 (2.2, 6.3)	8.8 (5.2, 12.4)
	*p*-value	0.382	0.235
Animal protein (g)	Egg	9.9 (8.0, 13.8)	18.1 (10.0, 26.0)
	Control	11.2 (8.1, 14.3)	15.6 (9.4, 22.8)
	*p*-value	0.349	0.255
Fat (g)	Egg	32.1 (29.5, 35.5)	37.5 (24.1, 45.3)
	Control	35.3 (29.6, 39.0)	33.2 (20.0, 40.3)
	*p*-value	0.070	0.151
Saturated fat (g)	Egg	14.0 (5.4, 15.2)	15.0 (6.5, 17.4)
	Control	14.2 (6.4, 15.8)	11.6 (5.4, 16.3)
	*p*-value	0.426	0.157
MU fat (g)	Egg	11.7 (4.3, 12.4)	12.9 (6.6, 15.6)
	Control	11.8 (4.8, 13.1)	10.7 (4.8, 14.2)
	*p*-value	0.195	0.075
PU fat (g)	Egg	3.6 (3.3, 4.2)	5.8 (4.1, 8.0)
	Control	3.6 (3.3, 5.6)	5.5 (3.2, 7.6)
	*p*-value	0.348	0.322
Cholesterol (mg)	Egg	94.6 (8.4, 101.5)	256.0 (90.5, 382.6)
	Control	94.5 (23.9, 105.7)	95.5 (27.8, 151.3)
	*p*-value	0.702	<0.001
Carbohydrates (g)	Egg	89.0 (75.4, 112.3)	126.5 (101.1, 145.4)
	Control	93.2 (75.6, 119.5)	130.5 (101.6, 159.0)
	*p*-value	0.330	0.336
Sugars (g)	Egg	52.3 (18.6, 61.5)	49.0 (30.9, 64.8)
	Control	53.4 (32.2, 61.9)	45.8 (23.9, 60.1)
	*p*-value	0.418	0.239
Fiber (g)	Egg	1.75 (1.16, 3.09)	4.96 (3.46, 7.74)
	Control	2.14 (0.99, 3.54)	5.60 (3.72, 9.05)
	*p*-value	0.664	0.217
Calcium (mg)	Egg	402 (314, 554)	380 (244, 532)
	Control	460 (325, 556)	380 (267, 578)
	*p*-value	0.404	0.787
Iron (mg)	Egg	6.51 (2.46, 9.35)	5.13 (3.27, 7.28)
	Control	5.95 (2.97, 9.79)	5.63 (3.40, 8.36)
	*p*-value	0.905	0.407
Magnesium (mg)	Egg	49.1 (29.8, 74.7)	112.6 (85.6, 149.7)
	Control	58.2 (38.5, 86.5)	122.9 (81.9, 159.8)
	*p*-value	0.046	0.444
Phosphorous (mg)	Egg	230 (161, 389)	460 (320, 637)
	Control	328 (199, 457)	447 (364, 673)
	*p*-value	0.068	0.880
Potassium (mg)	Egg	649 (458, 933)	881 (604, 1044)
	Control	764 (541, 1063)	875 (689, 1128)
	*p*-value	0.011	0.582
Zinc (mg)	Egg	3.27 (2.39, 5.10)	4.81 (3.31, 6.10)
	Control	3.46 (2.46, 5.34)	4.82 (3.65, 7.13)
	*p*-value	0.530	0.409
Copper (mg)	Egg	0.42 (0.36, 0.50)	0.57 (0.45, 0.71)
	Control	0.46 (0.39, 0.55)	0.55 (0.44, 0.67)
	*p*-value	0.083	0.560
Vitamin A (µg RE)	Egg	658 (516, 823)	552 (412, 762)
	Control	676 (523, 891)	530 (352, 752)
	*p*-value	0.428	0.567
Thiamin (mg)	Egg	0.51 (0.32, 1.00)	0.67 (0.46, 0.95)
	Control	0.61 (0.35, 0.87)	0.74 (0.49, 1.08)
	*p*-value	0.834	0.267
Riboflavin (mg)	Egg	0.60 (0.45, 0.93)	0.95 (0.60, 1.19)
	Control	0.71 (0.46, 1.08)	0.97 (0.59, 1.65)
	*p*-value	0.448	0.486
Niacin (mg)	Egg	3.67 (2.59, 5.80)	6.85 (4.48, 9.49)
	Control	4.03 (2.77, 5.82)	8.01 (5.82, 12.04)
	*p*-value	0.594	0.040
Vitamin B6 (mg)	Egg	0.43 (0.26, 0.59)	0.67 (0.49, 0.90)
	Control	0.47 (0.29, 0.72)	0.84 (0.60, 1.13)
	*p*-value	0.268	0.034
Folate (µg)	Egg	98.1 (59.9, 177.6)	143.7 (102.3, 235.7)
	Control	95.2 (63.5, 128.9)	157.1 (99.0, 233.1)
	*p*-value	0.437	0.769
Vitamin B12 (µg)	Egg	0.86 (0.50, 1.32)	1.63 (0.80, 2.43)
	Control	1.05 (0.63, 1.71)	1.18 (0.71, 1.87)
	*p*-value	0.402	0.199
Pantothenic acid (mg)	Egg	2.33 (1.83, 3.16)	3.20 (1.91, 4.42)
	Control	2.95 (2.03, 3.92)	2.72 (1.95, 4.31)
	*p*-value	0.167	0.545
Biotin (µg)	Egg	6.42 (2.29, 15.2)	14.82 (7.86, 25.22)
	Control	7.48 (2.00, 16.4)	11.17 (6.83, 16.02)
	*p*-value	0.810	0.070
Vitamin C (mg)	Egg	71.7 49.3, 92.1)	35.1 (26.6, 62.4)
	Control	67.3 (50.0, 99.8)	37.7 (29.4, 61.2)
	*p*-value	0.589	0.515
Vitamin D (µg)	Egg	3.27 (1.21, 6.57)	4.60 (1.00, 8.00)
	Control	4.25 (1.56, 6.67)	0.98 (0.64, 4.93)
	*p*-value	0.564	0.002
Vitamin E (mg)	Egg	3.61 (1.45, 8.91)	3.56 (2.12, 6.32)
	Control	3.27 (1.63, 7.07)	3.02 (1.64, 7.00)
	*p*-value	0.659	0.533

Values are medians, 25th and 75th percentile in parenthesis. MU, monounsaturated; PU, polyunsaturated; RE, retinol equivalents. ^1^ Baseline: egg group *n* = 63; control group *n* = 81; Endpoint: egg group *n* = 58; control group *n* = 74; ^2^ *p*-values in bold indicate significant differences between the egg group and control group, Mann–Whitney U test.

**Table 3:** Energy, macronutrient, and micronutrient intakes at endpoint for babies who ate egg during the 24-h recall period versus those who did not eat egg, regardless of treatment group.

Nutrient	Per 50 g Egg ^1^	Ate Egg (*n* = 47)	No Egg (*n* = 85)	*p*-Value ^2^
		Median (P25, P75)	Median (P25, P75)	
Energy (kcal)	308	985 (754, 1180)	899 (750, 1080)	0.282
Protein (g)	6.3	31.7 (23.7, 37.9)	22.3 (16.7, 29.0)	<0.001
Plant protein (g)	0	7.9 (5.1, 12.4)	7.8 (5.2, 10.8)	0.520
Animal protein (g)	6.3	23.7 (15.8, 30.3)	13.3 (6.9, 19.6)	<0.001
Fat (g)	5.1	38.2 (24.3, 49.9)	33.1 (19.4, 40.7)	0.026
Saturated fat (g)	1.5	15.0 (6.5, 18.1)	12.7 (5.0, 16.3)	0.069
MU fat (g)	2.0	13.55 (6.9, 15.9)	10.5 (4.4, 13.8)	0.006
PU fat (g)	0.7	6.6 (4.5, 9.3)	5.2 (3.3, 7.3)	0.005
Cholesterol (mg)	209.5	329.7 (277.6, 452.9)	86.1 (22.0, 108.6)	<0.001
Carbohydrates (g)	0.6	125.3 (92.4, 145.9)	131.9 (105.2, 156.0)	0.197
Sugars (g)	0.6	45.9 (15.9, 65.5)	47.6 (27.4, 60.3)	0.702
Fiber (g)	0.0	4.95 (3.48, 7.71)	5.53 (3.37, 8.93)	0.434
Calcium (mg)	19.5	393 (242, 562)	382 (267, 571)	0.847
Iron (mg)	0.9	5.77 (4.03, 7.64)	4.89 (2.68, 8.06)	0.197
Magnesium (mg)	4.5	123.0 (910.7, 151.2)	115.8 (83.0, 156.5)	0.657
Phosphorous (mg)	96	564 (393, 710)	428 (302, 580)	0.003
Potassium (mg)	49	938 606, 1159)	861 (682,1049)	0.529
Zinc (mg)	0.57	5.03 (3.72, 6.25)	4.59 (3.32, 6.68)	0.440
Copper (mg)	0.06	0.62 (0.43, 0.72)	0.53 (0.45, 0.662)	0.130
Vitamin A (µg RE)	33	600 (330, 890)	530 (374, 682)	0.216
Thiamin (mg)	0.05	0.72 (0.50, 0.94)	0.71 (0.46, 1.05)	0.934
Riboflavin (mg)	0.19	0.99 (0.73, 1.61)	0.84 (0.54, 1.42)	0.098
Niacin (mg)	0.05	7.53 (5.11, 0.16)	7.64 (4.73, 11.6)	0.926
Vitamin B6 (mg)	0.02	0.71 (0.56, 0.97)	0.78 (0.57, 1.11)	0.324
Folate (µg)	17.5	147.7 (103.1, 225.3)	156.5 (99.9, 243.5)	0.546
Vitamin B12 (µg)	0.8	1.98 (1.45, 3.37)	0.95 (0.49, 1.59)	<0.001
Pantothenic acid (mg)	0.74	3.81 (2.84, 4.88)	2.49 (1.79, 3.64)	0.001
Biotin (µg)	9.2	18.2 (13.7, 27.9)	8.4 (5.7, 14.3)	<0.001
Vitamin C (mg)	0	33.9 (16.3, 64.2)	37.7 (31.8, 59.6)	0.140
Vitamin D (µg)	3.97	5.37 (4.27, 8.84)	0.80 (0.62, 2.91)	<0.001
Vitamin E (mg)	1.73	4.78 (3.19, 8.05)	2.26 (1.42, 5.26)	<0.001

Values are medians, 25th and 75th percentile in parenthesis; 1 kcal = 4.186 kJ; MU, monounsaturated; PU, polyunsaturated; RE, retinol equivalents; ^1^ SAFOODS. SAMRC Food Composition Tables for South Africa [34]; ^2^ *p*-values indicate significance of differences between the babies who ate egg during the 24-h recall period versus those who did not eat egg, Mann–Whitney U test.

**Table 4:** Proportion of babies who consumed any foods from the eight foods groups during the 24-h recall period at baseline and endpoint.

Food Group	Group	Baseline (*n*) %	Endpoint (*n*) %
Breast milk	Egg	42 (66.7)	34 (38.6)
	Control	55 (67.9)	31 (41.9)
	*p*-value ^1^	0.875	0.056
Cereals, roots, and tubers	Egg	61 (96.8)	58 (100)
	Control	75 (92.6)	74 (100)
	*p*-value	0.271	-
Legumes	Egg	3 (4.8)	2 (3.4)
	Control	2 (2.5)	8 (10.8)
	*p*-value	0.8751	-
Dairy	Egg	42 (66.7)	45 (77.6)
	Control	55 (67.9)	60 (81.8)
	*p*-value	0.875	0.621
Flesh foods	Egg	4 (6.3)	24 (41.4)
	Control	7 (8.6)	37 (50.0)
	*p*-value	0.875	0.324
Egg	Egg	7 (11.1)	36 (62.1)
	Control	8 (9.9)	11 (14.9)
	*p*-value	0.810	<0.001
Vitamin A-rich fruit and vegetables	Egg	8 (12.7)	12 (20.7)
	Control	12 (14.8)	11 (14.9)
	*p*-value	0.716	0.965
Other fruit and vegetables	Egg	22 (34.9)	23 (39.7)
	Control	28 (34.6)	42 (56.8)
	*p*-value	0.965	0.051
Adequate DDS ^2^	Egg	5 (7.9)	21 (36.2)
	Control	5 (6.2)	14 (18.9)
	*p*-value	0.680	0.026
DDS (mean ± SD)	Egg	3.0 ± 0.9	4.0 ± 1.2
	Control	3.0 ± 0.9	3.7 ± 1.0
	*p*-value	0.939	0.094

DDS: dietary diversity score; ^1^ Fisher exact test; ^2^ five or more of the eight food groups.

**Table 5:** Foods consumed during the week preceding baseline, midpoint and endpoint, expressed as a percentage of babies for whom the food frequency questionnaire was completed.

Food Group	Food	Frequency of Consumption	Group ^1^	Baseline, %	Midpoint %	Endpoint %
Formula milk feeds	Formula milk	Every day	Egg	42.9	30.6	21.9
			Control	43.5	32.8	19.8
Dairy	Milk	≥4 days/week	Egg	10.0	12.9	18.8
			Control	8.2	17.9	25.9
	Yogurt	≥4 days/week	Egg	4.3	11.3	14.1
			Control	1.2	11.9	16.1
Baby foods	Pureed baby foods	≥4 days/week	Egg	20.0	29.0	7.8
			Control	23.5	17.9	5.0
	Infant cereal	≥4 days/week	Egg	61.4	46.8	7.8
			Control	65.7	26.9	6.2
Cereals, roots, tubers	Instant maize porridge	≥4 days/week	Egg	20.0	27.4	25.0
			Control	10.6	37.3	29.6
	Maize meal	≥4 days/week	Egg	14.3	48.4	65.6
			Control	20.0	55.2	72.8
	Porridge, other than maize	≥4 days/week	Egg	2.9	11.3	18.7
			Control	8.2	9.0	14.8
	Breakfast cereal	≥4 days/week	Egg	2.9	21.0	17.1
			Control	5.9	16.4	19.7
	Bread (commercial)	≥4 days/week	Egg	-	6.4	6.2
			Control	4.7	8.9	13.6
	Potato	≥4 days/week	Egg	14.3	35.5	17.2
			Control	14.1	19.4	18.5
Vegetables and fruit	Vegetables ^2^	≥4 days/week	Egg	4.3	9.7	4.7
			Control	-	6.0	9.9
	Fruit ^3^	≥4 days/week	Egg	4.3	16.1	7.8
			Control	4.7	13.4	12.3
Animal source foods	Chicken	≥1 day/week	Egg	32.8	71.0	79.7
			Control	34.1	61.2	82.7
	Meat	≥1 day/week	Egg	10.0	19.4	32.8
			Control	5.9	23.9	37.0
	Liver	≥1 day/week	Egg	25.7	35.5	43.8
			Control	30.6	35.8	48.1
	Fish	≥1 day/week	Egg	7.1	12.9	25.0
			Control	9.4	26.9	29.6
Unhealthy foods	Sweets	≥4 days/week	Egg	4.3	4.8	23.4
			Control	2.4	4.5	27.2
	Salty savory snacks	≥4 days/week	Egg	7.1	19.4	25.0
			Control	4.7	14.9	29.6
	Fizzy drinks	≥4 days/week	Egg	1.4	4.8	9.4
			Control	2.4	7.5	18.5
	Cordials (mix with water)	≥4 days/week	Egg	-	8.1	14.0
			Control	2.4	3.0	8.6

^1^ Baseline: egg group *n* = 70; control *n* = 85; Midpoint: egg group *n* = 62; control *n* = 67; Endpoint: egg group *n* = 64 control *n* = 81; ^2^ Vegetables eaten by at least 20% of babies: Cabbage (midpoint 26.4%, endpoint 24.8%); carrot (baseline 21.9%, midpoint 27.9%, endpoint 24.8%); pumpkin (baseline 43.2%, midpoint 48.8%, endpoint 35.2%); ^3^ Fruit eaten by at least 20% of babies: Apple (endpoint 48.3%); banana (baseline 33.5%, midpoint 36.4%, endpoint 46.0%); orange (midpoint 31.8%).

**Table 6:** Consumption of eggs during the preceding week, expressed as a percentage of babies for whom the food frequency questionnaire was completed.

Food	Group ^1^	Frequency of Consumption	Baseline %	Midpoint %	Endpoint %
Egg consumption during the previous week	Egg	Every day	1.4	87.1	53.1
		4–6 days	4.3	8.1	21.9
		1–3 days	41.4	4.8	15.6
		Never	52.9	0	9.4
	Control	Every day	4.7	9.0	4.9
		4–6 days	4.7	10.4	9.9
		1–3 days	35.3	41.8	45.7
		Never	55.3	38.8	39.5

^1^ Baseline: egg group *n* = 70; control *n* = 85; Midpoint: egg group *n* = 62; control *n* = 67; Endpoint: egg group *n* = 64; control *n* = 81.

**Table 7:** Preparation of eggs and amount eaten, expressed as a percentage of babies who ate egg during the preceding week.

Food	Group ^1^		Baseline %	Midpoint %	Endpoint %
Preparation	Egg	Boiled	48.5	29.0	41.4
		Scrambled	18.2	24.2	19.0
		Fried	33.3	46.8	39.7
	Control	Boiled	34.2	19.5	16.3
		Scrambled	21.1	19.5	16.3
		Fried	44.7	61.0	63.3
Food items added during preparation	Egg	Oil	30.3	29.0	31.0
		Margarine	18.2	32.3	20.7
	Control	Oil	30.3	46.3	57.1
		Margarine	18.2	31.7	18.4
Mix egg with other food ^2^	Egg	Yes	9.1	75.8	51.7
	Control	Yes	23.6	40.5	32.7
Portion size usually eaten	Egg	>1 egg	-	-	13.8
		1 egg	60.6	80.6	86.2
		½ egg	24.2	16.1	-
		¼ egg	15.2	3.2	-
	Control	>1 egg	-	-	20.4
		1 egg	50.0	78.0	69.4
		½ egg	44.7	17.1	10.2
		¼ egg	2.6	2.4	-
		<¼ egg	2.6	2.4	-
Number of times egg eaten per day	Egg	Once	87.9	87.1	84.5
		2 times	9.1	9.7	12.1
		3 times	3.0	3.2	3.4
	Control	Once	84.2	66.7	83.7
		2 times	10.5	26.2	14.3
		3 times	5.3	4.8	2.0

^1^ Baseline: egg group *n* = 33; control *n* = 38; Midpoint: egg group *n* = 62; control *n* = 41; Endpoint: egg group *n* = 58; control *n* = 49; ^2^ Egg was mixed mostly with porridge and potato; some ate egg with bread.

**Table 8:** Respondents’ perceptions regarding the benefits and risks of giving egg to the baby, expressed as a percentage of respondents who completed the exit interview.

		Total Group %
Benefits of giving egg to baby	Improves growth	22.3
	Improves weight	12.9
	Improves health	10.8
	Provides protein	28.8
	Provides vitamins/nutrients	8.7
	Provide energy	2.9
	Other ^1^	6.5
	Don’t know	7.2
Risks of giving egg to baby	None	95.2
	May cause allergy	3.4
	Other	1.4
Best time to start giving egg to baby	Before 6 months	30.1
	6 months	54.5
	After 6 months	15.4

^1^ Range of answers: Baby enjoys it, improves appetite, improves memory, baby will be clever, good for the skin, food for baby when there was no food available in household.

**Table 9:** Frequency of egg intake in relation to incidence and duration of allergy-related symptoms of babies during the 8-month intervention ^1^.

Allergy-Related Symptoms	Incidence, n (%) Duration, Median (25th, 75th)			Association with Frequency of Egg Consumption	
				Unadjusted	Adjusted ^2^
	Total (*n* = 155)	Egg Group (*n* = 70)	Control Group (*n* = 85)	Exp B 95% CI)/ β *p*-Value ^1^ (*n* = 155)	Exp B 95% CI)/ β *p*-Value ^1^ (*n* = 155)
Possible allergy reported by caregiver	29 (4)	10 (3.0)	19 (4.4)	1.049 (0.896, 1.227); 0.553	0.790 (0.804, 1.180); 0.790
Eczema	15 (2)	7 (2.1)	8 (1.9)	0.991 (0.796, 1.234); 0.939	0.875 (0.667, 1.147); 0.334
Eczema duration (d)	5 (3, 8)	6 (3, 12)	7 (4, 50)	0.234; 0.489	0.836, 0.102
Other rash	9 (1)	4 (1.2)	5 (1.2)	1.119 (0.849, 1.474); 0.426	1.105 (0.752, 1.626); 0.611
Other rash duration (d)	7 (3, 11)	8 (4, 13)	3 (2, 11)	−0.294; 0.443	−0.269; 0.797
Angioedema	2 (0.3)	1 (0.3)	1 (0.2)	0.961 (0.527, 1.754); 0.898	0.896 (0.472, 1703); 0.737
Wheeze	31 (4)	14 (4.2)	17 (3.9)	0.969 (0.830, 1.132); 0.690	0.978 (0.806, 1.185); 0.817
Wheeze duration (d)	11 (4, 28)	4.5 (3, 16)	15 (7, 28)	0.413; **0.021**	−0.155; 0.580

^1^ Association of frequency of egg intake with incidence and duration of symptoms were tested with logistic and linear regression, respectively. ^2^ Adjusted for age, sex, birth weight, frequency of breastfeeding and formula intake, total energy intake, and education level of the caretaker.

## Data Availability

As required by the Health Research Ethics Committee of the North-West University, South Africa, the researchers signed confidentiality agreements in which they agreed ‘not to disclose confidential information to anyone other than North-West University unless required to do so by law or by court order’. Participant consent forms were also clear for the purposes of data usage. Permission to obtain the data can be obtained from the North-West University Health Research Ethics Committee, Prof Wayne Towers: wayne.towers@nwu.ac.za.
